# Using XGBoost and SHAP to explain citizens’ differences in policy support for reimposing COVID-19 measures in the Netherlands

**DOI:** 10.1007/s11135-024-01938-2

**Published:** 2024-07-24

**Authors:** Jose Ignacio Hernandez, Sander van Cranenburgh, Marijn de Bruin, Marijn Stok, Niek Mouter

**Affiliations:** 1 Center of Economics for Sustainable Development (CEDES), Faculty of Economics and Government, Universidad San Sebastian, Concepción, Chile; 2 Transport and Logistics Group, Faculty of Technology, Policy and Management, Delft University of Technology, Delft, Netherlands; 3 National Institute of Public Health and the Environment, Bilthoven, Netherlands; 4 Institute of Health Sciences, IQ Healthcare, Radboud University Medical Center, Nijmegen, Netherlands; 5 Department of Interdisciplinary Social Science, Utrecht University, Utrecht, Netherlands; 6 Populytics, Research Agency, Frambozenweg, 136 2321 KA, Leiden, Netherlands

**Keywords:** XGBoost, SHAP, Policy support, COVID-19, SARS-CoV-2, Participatory Value Evaluation

## Abstract

Several studies examined what drives citizens’ support for COVID-19 measures, but no works have addressed how the effects of these drivers are distributed at the individual level. Yet, if significant differences in support are present but not accounted for, policymakers’ interpretations could lead to misleading decisions. In this study, we use XGBoost, a supervised machine learning model, combined with SHAP (Shapley Additive eXplanations) to identify the factors associated with differences in policy support for COVID-19 measures and how such differences are distributed across different citizens and measures. We use secondary data from a Participatory Value Evaluation (PVE) experiment, in which 1,888 Dutch citizens answered which COVID-19 measures should be imposed under four risk scenarios. We identified considerable heterogeneity in citizens’ support for different COVID-19 measures regarding different age groups, the weight given to citizens’ opinions and the perceived risk of getting sick of COVID-19. Data analysis methods employed in previous studies do not reveal such heterogeneity of policy support. Policymakers can use our results to tailor measures further to increase support for specific citizens/measures.

**Supplementary Information:**

The online version contains supplementary material available at 10.1007/s11135-024-01938-2.

## Introduction

The outbreak of the COVID-19 pandemic forced governments to strategically adopt measures to control multiple waves of the virus. With new variants of SARS-CoV-2 appearing (e.g., Alpha, Delta, Omicron), governments faced a trade-off between different measures that could prevent new infections, avoid further deaths due to COVID-19 and reduce the risk of overloading the healthcare system. However, such measures would also increase psychological stress and impact the economy, which in turn would hinder the citizens’ support and decrease adherence. By understanding what factors explain the citizens’ policy support (i.e., the extent that citizens agree with conducting specific policy options) for COVID-19 measures, governments can prioritise those measures that are effective in curbing the spread of the virus and, at the same time, are widely accepted.

Previous studies shed light on the factors that explain the support for COVID-19 measures, mostly by using descriptive statistics, regression analysis, discrete choice modelling and Latent Class Cluster Analysis (LCCA). These studies conclude, for instance, that higher policy support for COVID-19 measures is associated with the citizens’ trust in institutions (Dohle et al. 2020; Gotanda et al. 2021), perceived risk, sociodemographic characteristics (Mouter et al. 2022; Sicsic et al. 2022) and geographical factors (Loria-Rebolledo et al. 2022). However, in most of these works, the existence of (observed) heterogeneity of preferences across respondents is barely studied or, in some cases, overlooked because their data analysis methods can only explain the policy support in terms of “average” effects. For instance, regressions and discrete choice models provide outcomes that are interpretable for a representative citizen or specific measure, while LCCA identifies different groups of citizens and characterises them in terms of averages within each group. In all cases, the effects are “averaged-out” in different degrees. This could lead researchers to overlook potential differences in preferences across specific citizens or measures that, if substantial, can lead to misguided policy advices.

To overcome these limitations, supervised machine learning (ML) models can be used. A supervised ML model aims to predict one or more response variables (e.g., whether an individual accepts COVID-19 measures) in terms of a set of covariates. Among specific supervised ML models, XGBoost can learn complex interactions between covariates and individual effects without the need of being previously specified by the analyst, reaching a high prediction performance and, at the same time, overcoming the limitations of previous studies to explain the policy support for COVID-19 measures. But like many other ML methods, XGBoost only provides an overall importance level of each covariate for predicting the response variable (i.e., a global explanation), making this ML method relatively ‘opaque’ in terms of explainability. So-called explainable AI (XAI) methods can overcome this limitation of XGBoost. XAI methods aim to provide explanations from an otherwise ‘opaque’ ML model. An XAI method that gained popularity nowadays in literature is SHAP. This method relies on coalitional game theory to provide local explanations (i.e., at the individual level). The idea behind SHAP is to explain how the response of each individual did deviate from the average response, in terms of a set of covariates (e.g., sociodemographic characteristics, experimental features, etc.). This approach is similar to other XAI methods, namely LIME or LRP, but the advantage of SHAP their rooting in a formal theory, which provides this method with a greater robustness and makes it more trustable for its use in policy applications. Therefore, using XGBoost and SHAP to explain the policy support for COVID-19 measures allow researchers to, for instance, explain how the differences in policy support are distributed across respondents, spot non-linear effects that could be overlooked by conventional methods, or explain responses of specific profiles of respondents that are of the interest of policymakers. Furthermore, such information can be used by policymakers to tailor policies for specific citizens in order to increase their acceptance for specific COVID-19 measures in future pandemics.

This paper aims for two goals. Firstly, we explore the extent that XGBoost combined with SHAP can explain the differences in policy support for COVID-19 measures at the respondent level. Secondly, we explore the extent that SHAP differs from conventional data analysis methods used in previous studies, namely choice models and LCCA, in terms of their degree of detail, interpretation and technical aspects (e.g., statistical significance, estimation time). To reach these goals, this paper makes use of a dataset originally reported by Mouter et al. (2022) from a Participatory Value Evaluation (PVE) experiment conducted in the Netherlands to infer the Dutch citizens’ preferences for reimposing a set of COVID-19 measures under different risk scenarios.

## Experiment and data

This paper makes use of a dataset from a PVE experiment. PVE experiments have been applied in diverse fields, including COVID-19 measures (Mouter, Hernandez, et al., 2021), healthcare investments (Mulderij et al. 2021; Rotteveel et al. 2022) and public infrastructure projects (Mouter, Koster, et al. 2021a, b). In a PVE experiment, respondents are asked to imagine a certain scenario and then choose a combination of policy alternatives for addressing the scenario.

In the PVE experiment of Mouter et al. (2022), four different scenarios were designed, describing different levels of COVID-19 threat and the current hospital overcrowding risk (see Table 1).

**Table 1:** Description of scenarios of the PVE experiment, based on Mouter et al. (2022)

	Scenario description	Number of possible COVID-19 measures	Initial hospital overcrowding risk
Scenario 1	Hospitalizations are at a low level. No operations are postponed. There is no dangerous new variant of the virus	9	45%
Scenario 2	Autumn has begun, and COVID-19 spread faster. Hospitalisations of vulnerable people and non-vaccinated increase. Minor surgeries are postponed. Basic rules are imposed (i.e., wash your hands, keep 1.5 m distance and get tested in case of symptoms)	14	69%
Scenario 3	A new variant that spreads faster is found in another country, but it is not clear whether this variant generates more severe symptoms. Restrictions for entering the country are imposed, as well as the basic rules. There is a risk that major surgeries in hospitals will be postponed	14	60%
Scenario 4	A new variant is found in another country, which spreads faster, and it is clear that many people have severe symptoms from this variant. In addition to the basic rules and entry restrictions, more severe measures are in place (e.g., capacity limits to the hospitality industry and no massive events). The healthcare capacity is at its limits, and if no measures are taken, major surgeries will be postponed, and there is a risk that patients will no longer be able to go to a hospital	13	100%

Each scenario was embedded in an independent PVE experiment choice task. For every scenario, a list of possible policy alternatives was presented. By choosing a policy alternative, the hospital overcrowding risk is reduced in a specific percentage within predefined ranges (see Table 2), defined in consultation with healthcare experts (Mouter et al. 2022). In scenarios 1, 2 and 3, respondents were allowed to choose any combination of policy alternatives, whereas in scenario 4, they must choose a combination that results in at least a 30% reduction in the hospital overcrowding risk. Each respondent answered three out of four scenarios: scenarios 1 and 2 are answered by all respondents, and scenarios 3 and 4 are randomly assigned to each respondent.

**Table 2:** COVID-19 measures per scenario, adapted from Mouter et al. (2022)

Scenarios					
Measures	Risk red. range	S1	S2	S3	S4
Advice to wash hands frequently and thoroughly	1–3	X			
Advice to stay at home with COVID-19 symptoms and to do a test	3–5	X			
Advice not to shake hands	8–14	X			
Advice to ventilate	3–7	X			
Advice to keep 1.5 m distance	7–13	X			
Quarantine if in intensive contact with person infected with COVID-19	4–8	X			
Advice to work at home a few days a week	2–4	X			
Advice to work at home, unless it is absolutely necessary	6–10		X	X	X
Mouth mask obligation in public transport/shops/hospitality industry	2–6	X	X	X	
Vaccination passport hospitality industry (2G or 3G)	3–5	X	X	X	
Vaccination passport for people working with vulnerable people	5–8		X	X	X
Vaccination passport except in schools, work and essential shops	4–10		X	X	X
Encourage self-testing by making it available free of charge	6–10		X	X	
Starting a booster campaign which starts with vulnerable people	10–15		X	X	X
Requiring shops to offer time slots for people with vulnerable health	5–8		X	X	
Limit number of customers per square metre in non-essential shops	1–3		X		
Pick up orders in non-essential shops	2–4		X	X	X
1/3 capacity and fixed seating at events	2–6		X	X	
Banning festivals and major sporting events	4–8		X	X	
Strict advice not to have more than 2 visitors per day at home	5–10		X	X	X
Advice higher education online and maximum number of students per college	4–8		X	X	X
Lockdown after 5 pm	8–10			X	
Lockdown after 8 pm	4–8			X	X
Closing restaurants/cafés	10–15				X
Closing sports venues	5–10				X
Closing cinemas, theatres, concert halls	5–10				X
Closing primary/secondary schools	15–20				X

**Fig. 1:**
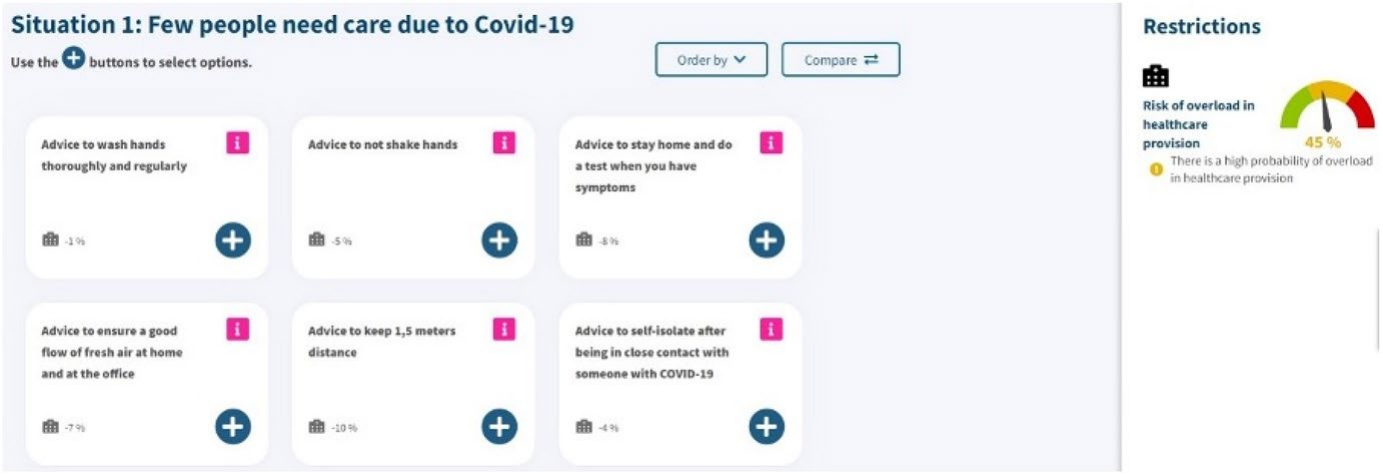
Example choice task presented in the PVE experiment for scenario 1

The PVE experiment choice tasks were embedded in a web survey. After the presentation of an instruction video, respondents were presented with the PVE choice tasks (see an example in Fig. 1). Policy alternatives with their respective reductions of the hospital overcrowding risk are presented in the left-side pane, whereas the total hospital overcrowding risk is detailed in the right-side pane as an interactive gauge. After answering the choice tasks, respondents have to fill out a questionnaire about their sociodemographic profile (e.g., gender, age, living province) and perception questions (e.g., perceived risk of being affected by a COVID-19 infection, the weight they believe governments should give to scientists or citizens’ opinion, etc.)

The data was collected between 3 and 10 February 2022 and corresponds to a representative panel collected by a specialised survey company (Mouter et al. 2022). After cleaning missing values and no responses, the final dataset used for this paper comprises 5,664 responses from 1,888 respondents (since each respondent answered three choice tasks) and 15 variables (see Table 3).

**Table 3:** Variables used in this study

Variable	Description	Values
*Experimental features*		
Overcrowding risk reduction	Reduction of risk of overloading the healthcare system (%) derived from each specific COVID-19 measure	Numeric (see Table 2)
*Sociodemographic characteristics*		
Gender	Gender of the respondent	Male
		Female
Age	Age group	18–24 years
		25–34 years
		35–44 years
		45–54 years
		55–64 years
		65–74 years
		75 or more years
Education	Education level	Low
		Medium
		High
Province	Living province	Categorical per province
City size	Size category of the living city of respondents	Village
		Small city
		Medium city
		Big city
Work status	Type of work/work status	Full-time
		Part-time
		Retired
		Incapacitated
		Student
		Unemployed
*Vaccination status*		
Vaccinated	Whether the respondent has at least one COVID-19 vaccine	Binary (No/Yes)
Boosted	Whether the respondent has a booster shot	
*Perception indicators*		
Risk (infected)	Perceived risk of getting infected by COVID-19	No risk
Risk (getting sick)	Perceived risk of getting very sick by COVID-19	Small risk
Risk (hospitalised)	Perceived risk of getting hospitalised by COVID-19	Moderate risk
Risk (death)	Perceived risk of dying by COVID-19	High risk
Weight citizens' opinion compared to scientists' opinion	The weight that a respondent believes the government should put on the opinion of citizens in this survey with respect to the opinion of scientists and experts. Higher values indicate a higher value to the citizens’ opinion	Only to citizens
		Citizens more than scientists
		Citizens equally to scientists
		Scientists more than citizens
		Only scientists
*Response variables*		
Choice	Response variables per measure. They indicate if a specific COVID-19 measure was chosen in the scenario	Binary (No/Yes)

We considered 14 covariates, based on previous studies including the original work of Mouter et al. (2022). We distinguish between four covariates types: experimental features, sociodemographic characteristics, vaccination status, and perception indicators. Regarding the experimental features, we include the overcrowding risk reduction of each COVID-19 measure. The sociodemographic characteristics considered in this study are the respondents’ gender, age group, education level, living province, city size and work status. The vaccination status is divided into two covariates: whether the respondent is vaccinated at least once, and whether they received a booster shot. The first set of perception indicators considered in this study is the respondent’s perceived risk that their health would be affected by COVID-19 in four levels: getting infected by the virus, getting very sick, being hospitalised and dying due to the disease. The final perception indicator is the respondent’s weight they think the government should give to the citizens’ opinion, relative to the scientists’ opinion. Finally, the response variables (Choice) are binary variables equal to one if a COVID-19 measure was chosen by the respondent and zero otherwise. Each measure is associated with an independent response variable, and the response variables for the same measure are independent across scenarios. Furthermore, the response variables are not mutually exclusive. Therefore, the policy support for COVID-19 measures consist of the extent that citizens agree/disagree with conducting each measure on each scenario of the PVE experiment.

## Methods

Data is analysed using XGBoost (Chen & Guestrin 2016), a supervised ML model of the family of tree-boosting models. XGBoost was chosen among alternative ML models (i.e. neural networks and random forests) since tree-boosting models have been proven to be robust to overfitting and, furthermore, reaching higher predictive performance in choice data (Wang et al. 2021).^1^ After the model is trained, SHAP is applied on it to uncover what relations has been learned from the data and explain the differences on the predicted the policy support for COVID-19 measures, measured as the predicted probability of choosing such measure for each respondent. Finally, the outcomes of SHAP are visualised and interpreted.

The following subsections describe XGBoost, SHAP and the use of their outcomes.

### XGBoost

XGBoost is a ML system for tree-boosting. Tree-boosting is an algorithm that combines the outcomes of a set of decision tree (DT) models to form a model with higher predictive performance. A DT is a ML model that predicts one or more response variables contained in $Y$ as a set of conditions that the set of covariates $X$ must hold, forming a tree structure. Given $Y$ and a set of covariates $X$, the tree-boosting algorithm aims to predict $Y$ as detailed in Eq. (1):

1 $$ \hat{Y} = \hat{f}_{T} \left( X \right) = \sum\limits_{{t = 1}}^{T} {\hat{f}_{t} \left( X \right)} , $$

where $T$ is the number of DT models, ${\widehat{f}}_{T}$ is the tree-boosted model and ${\widehat{f}}_{t}$ is the t-th DT model. In the tree-boosting algorithm, each DT model is added sequentially. On each iteration, the new DT model corrects the mispredictions of the tree-boosted model that is formed thus far. Mathematically, the tree-boosting algorithm optimises a loss function $l(\cdot )$ that depends on ${Y}_{i}$ at a step $t$ and the predictions ${{\widehat{Y}}_{i}}^{(t-1)}$ of the previous $t-1$ models, plus a regularisation term $\Omega (\cdot )$. On each step $t$ of the tree-boosting algorithm, the overall loss function can be written as in Eq. (2):

2 $${L}^{(t)}={\sum }_{i=1}^{N}l({{\widehat{Y}}_{i}}^{(t-1)},{Y}_{i})+{\sum }_{t=1}^{T}\Omega (\widehat{{f}_{t})}.$$

XGBoost is a form of gradient-boosting (Friedman 2001) in which the objective function depends on the learning problem (e.g., classification, regression, etc.), the loss function that is optimised in XGBoost changes. Our implementation of XGBoost is for a multi-label classification problem, since the responses of the PVE experiment are binary, non-mutually exclusive variables. Therefore, the output of XGBoost is a vector of probabilities of choosing each of the COVID-19 measures, independently.

Given the learning problem of our implementation, the objective function and evaluation metric of XGboost are a logistic and log-loss functions, respectively (see Table 4). In addition, we optimised three hyperparameters using a grid search process, in which each possible combination of hyperparameters are used to train the XGBoost model using a tenfold cross validation. The average loss is computed and the final model is the one for which the average loss is minimum. For all scenarios, the optimal hyperparameters are a Gamma value equal to 2, a maximum tree depth equal to 3 and a minimum child weight equal to 5.

**Table 4:** Hyperparameters used in XGBoost

Hyperparameter	Description	Candidate Values
Loss function	The objective loss function to optimise	binary:logistic (fixed)
Evaluation metric	Metric evaluated on each iteration as stopping criterion of the optimisation algorithm	logloss (fixed)
Gamma	Minimum loss reduction for making a partition of the tree models. A higher value implies a higher regularisation at the risk of underfitting	0, 1, 2 (optimal = 2)
Maximum tree depth	Maximum number of levels of the tree models. Higher depth can capture more interactions, at the risk of overfitting the final model	3, 5, 7 (optimal = 3)
Minimum child weight	Minimum sum of weights of a child node. Higher values prevent the algorithm to make too much splits on the trees, at the risk of underfitting the final model	1, 2, 3, 5 (optimal = 5)

After selecting the optimal hyperparameters, the training process was done using a combination of tenfold cross validation and a split sample. On each scenario, a random split of the data is done: 80% of the sample is used for training, and the remaining 20% is left as a holdout (test) sample. The training process is performed using tenfold cross validation using the training sample only. After the model is trained, the SHAP values are computed for the holdout sample.

### SHAP

SHAP (Lundberg et al. 2017) is a technique to provide explanations for an otherwise “opaque” ML model. SHAP calculates how much each covariate contributes to the prediction of each respondent of the sample with respect to the average prediction in terms of Shapley values. Shapley values are a concept of coalitional game theory that describes the distribution of payments across coalitions of players in a cooperative game.

While SHAP has gained increasing popularity in the ML field, its use for choice problems has been rather minor and recent. A brief literature review shows that the use of SHAP to address choice problems has been scoped mostly in the transportation field (e.g., Dong et al. 2022; Ji et al. 2022; Jin et al. 2022; Lee 2022). For instance, Dong et al. (2022) use SHAP in an artificial neural network to explain individual and general route choice behaviour from GPS data in South Korea; Ji et al. (2022) applies SHAP in an XGBoost model to uncover interactions between covariates that explain Cyclists’ behaviour in China; Jin et al. (2022) compares the explanations from gradient-boosting methods and SHAP with the interpretations of a multinomial logit model to explain vehicle transactions in the United States; and Lee (2022) uses SHAP and XGBoost to explain the decision of giving up the use of public transport during the COVID-19 pandemic in South Korea. To the authors’ knowledge, the only applications of SHAP outside the transportation field are Wang et al. (2022), who use SHAP and a series of ML models (e.g., random forests, neural networks, XGBoost) to explain the decision of getting online healthcare in China, and this work.

SHAP relates ML with game theory by assuming that a set of covariates ${X}_{n}=\{{x}_{n1},{x}_{n2},\dots \}$ for a specific respondent $n$ are players in a game that consists of predicting the response variable ${Y}_{n}$. The game is the ML model, and the payoffs are the predictions $\widehat{f}({X}_{n})$. Each covariate can contribute to the prediction standalone or forming a coalition with one or more other covariates. The Shapley value ${\phi }_{nk}$ of a covariate value ${x}_{nk}$ for a respondent $n$ is the averaged marginal contribution of ${x}_{nk}$ to predict ${Y}_{n}$, across all possible coalitions (Molnar 2020), given by Eq. (3):

3 $${\phi }_{nk}={\sum }_{S\subset \{1,\dots ,K\}\backslash \{k\}}\frac{|S|(K-|S|-1)!}{K!}({\widehat{f}}_{x}(S\cup k)-{\widehat{f}}_{x}(S)),$$

where $S$ is a subset of the covariates of the model, $K$ is the number of covariates, and ${\widehat{f}}_{x}(S)$ is the prediction for the covariates in set $S$ marginalised over the covariates that are not included in $S$.

The outcome of SHAP is a matrix $N\times K$ of SHAP values, computed per response variable. In other words, SHAP values are computed at each respondent’s level, per covariate and per response variable (i.e., per COVID-19 measure). There are multiple algorithms to compute SHAP values. In our implementation, we use the so-called Exact Explainer, in which actual Shapley values are computed through enumeration.

SHAP values satisfy the properties of local accuracy, missingness and consistency (Lundberg et al. 2017). Local accuracy guarantees that the sum of SHAP values for a respondent $n$ is equal to the difference between the prediction for $n$ and the average prediction across all respondents. Missingness guarantees that if a covariate value ${x}_{nk}$ is missing, then its SHAP value is zero, thus not affecting the local accuracy property. Consistency guarantees that if the contribution of ${x}_{nk}$ increases, then its SHAP value also increases.

SHAP presents three key advantages over alternative XAI methods, such as the Local Interpretable Model-Agnostic Explanations (LIME) proposed by Ribeiro et al. (2016) and Layer-Wise Relevance Propagation (LRP) proposed by Bach et al. (2015). Firstly, SHAP bases its explanations on computing Shapley values, which makes this method theoretically robust and stable compared to LIME and LRP, which base their explanations on random perturbations over the dataset. Secondly, SHAP is model agnostic, similar to LIME, but different from LRP, which is specific to neural networks. Therefore, SHAP can be used on any supervised ML model. Thirdly, SHAP allows for both local and global explanations, since the computed Shapley values can be aggregated (i.e., averaged) to explain the mean contribution of each covariate.

### Using the outcomes of SHAP: SHAP importances and visualising SHAP values

SHAP values are used in two forms (see Table 5). Firstly, we compute so-called SHAP importances. The SHAP importance of a covariate is the absolute value of its associated SHAP values averaged across respondents and policies of a specific scenario, as shown in Eq. (4):

**Table 5:** Summary of approaches to interpret SHAP values used in this paper

	SHAP importances	Visualisation of SHAP values
Definition	The absolute SHAP values averaged across respondents	The SHAP values associated with a covariate, presented in plots
Information that provides	The average effect of a specific covariate on the policy support for COVID-19 measures	The distribution (sparsity) of the effects of specific effects and nonlinear effects
Type of interpretation	Numerical [0,1]	Visual (plots)
Meaning	If low: The covariate has a *small* effect on the policy support for COVID-19 measures, on average	Summary plot: It plots the SHAP values per covariate, sorted by their importance. Each plot shows the *distribution of the effects* associated with a covariate for the policy support for a specific COVID-19 measure. Each point is coloured according to its associated covariate value
	If high: The covariate has a *great* effect on the policy support for COVID-19 measures, on average	Scatter plot: It plots the SHAP values for a specific covariate. It allows for identifying *nonlinear effects* in the policy support for a specific COVID-19 measure
		Waterfall plot: It plots the SHAP values of a specific respondent for a given COVID-19 measure. It allows to explain specific citizen profiles in terms of covariates

4 $${\overline{\phi }}_{k}=\left|\frac{{\sum }_{n=1}^{N}{\phi }_{nk}}{N}\right|,$$

SHAP importances are bound between 0 and 1 since its associated SHAP values represent variations of the probability of choosing specific policies from the average response, in a specific scenario. Higher (lower) SHAP importances indicate that, on average, a covariate has a greater (smaller) effect on the policy support for COVID-19 measures. Thus, the analyst should prioritise interpreting covariates with high SHAP importances.

SHAP importances are a form to provide global explanations from SHAP values, and they are comparable to the variable importances of XGBoost. However, SHAP importances measure the average deviation of a covariate from the average response, as a difference from the variable importances of XGBoost, which are the average contribution that each variable’s split point improves the performance measure used during training. In consequence, the interpretation of SHAP importances is more related to variations on policy support than the native importances of XGBoost.

It is important to notice that a low SHAP importance does not necessarily mean that a covariate has a negligible effect, but it means that such effect is smaller than the effect of other covariates. Hence, we use SHAP importances to identify the three most relevant covariates in all three risk scenarios, to focus the visualisation and interpretation of SHAP values in this paper. A detailed visualisation of all covariates per scenario is presented in supplementary material 1.

After the three most important covariates are identified, SHAP values are visualised in three specific plots to facilitate their interpretation. The first visualisation is the so-called summary plot. Given a specific covariate, a summary plot details how much the SHAP values are distributed across respondents in terms of magnitude and direction. Each point of the summary plot is the SHAP value of a specific respondent associated with a specific covariate. The horizontal axis details the magnitude of the SHAP value. If two SHAP values are of similar value, they are stacked vertically, showing observed homogeneity/heterogeneity of effects for different respondents. Specifically, a summary plot with SHAP values with higher height indicates a group of respondents with homogeneous policy support for the associated COVID-19 measure, whereas a plot with a lower height (or resembling a line) indicates few respondents with similar policy support for COVID-19 measures. Finally, SHAP values are coloured according to the covariate values to detail the direction of the effects of each covariate.

The second visualisation is scatter plots of the SHAP values for a specific COVID-19 measure and covariate. Scatter plots detail the relationship between a specific covariate with its associated SHAP values. The vertical axis of the scatter plot details the magnitude of the SHAP values associated with a specific covariate, whereas the horizontal axis details the values of such covariate. SHAP scatter plots allow analysts to identify how the effects on the policy support for a COVID-19 measure are distributed across the values of a specific covariate. From a scatter plot, the analyst can identify nonlinear effects or specific effects per groups of respondents.

The third visualisation is so-called waterfall plots for the SHAP values of a specific respondent and COVID-19 measure. Given a specific respondent (hence, a vector of specific covariate values), waterfall plots detail how much each covariate did contribute (positively or negatively) from the average probability of choosing a COVID-19 measure to the predicted probability of a specific respondent. Hence, waterfall plots can be used to explain the responses of specific citizens profiles, in terms of the covariates used to fit the XGBoost model.

## Results

### SHAP importances

We compute the SHAP importances per risk scenario, averaged across respondents and COVID-19 measures (see Table 6). In addition, the average SHAP importance across risk scenarios is calculated (last column) to identify which covariates are the most (least) important across scenarios, on average.

**Table 6:** SHAP importances per risk scenario

	Scenario 1	Scenario 2	Scenario 3	Scenario 4	*Average*
Gender	0.013	0.011	0.019	0.011	0.014
Age	**0.032**	**0.025**	0.023	**0.029**	0.027
Education	0.017	0.015	0.018	0.009	0.015
Province	0.022	0.019	0.021	**0.021**	0.021
City size	0.008	0.011	0.014	0.015	0.012
Work status	0.021	0.018	**0.026**	0.019	0.021
Vaccinated	0.021	0.016	0.014	0.011	0.016
Boosted	0.013	0.024	0.023	0.013	0.018
Risk (infected)	0.015	0.010	0.012	0.012	0.012
Risk (getting sick)	**0.029**	**0.026**	**0.027**	0.013	***0.024***
Risk (hospitalised)	0.011	0.013	0.016	0.020	0.015
Risk (death)	0.019	0.015	0.019	0.017	0.017
Weight citizens/scientists opinion	**0.027**	**0.032**	**0.039**	**0.021**	***0.030***
Overload risk reduction	0.011	0.008	0.012	0.012	*0.011*

The filling intensity details a higher importance per scenario. The three most relevant covariates are in bold

On average, the most important covariates are, in descending order, the weight of citizens'/scientists' opinion, age and the perceived risk of getting sick of COVID-19. These three covariates are also the most important in all scenarios, except in scenario 3, where work status becomes the third-most important covariate. On the other hand, the overcrowding risk reduction generated by the measures is consistently ranked as one the least important covariates. These results indicate that sociodemographic characteristics and perception indicators explain better the differences in the policy support for COVID-19 measures than the resulting reductions in the risk of overloading the healthcare system. In the following subsections, we focus on the visualisation of SHAP values of age, the perceived risk of getting sick of COVID-19 and the weight of citizens'/scientists' opinion.

### Visualising SHAP values

Now, we present visualisations of the SHAP values for the three most important covariates, namely the age group, the weight of citizens'/scientists' opinion and the perceived risk of getting sick of COVID-19. A complete set of summary plots per covariate, measure and scenario is provided in supplementary material 1.

#### Age group

**Fig. 2:**
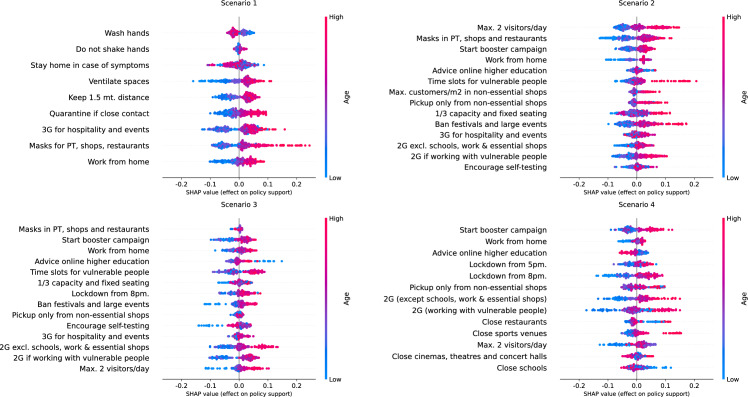
SHAP summary plots of age group, per measure and risk scenario

We generate summary plots of the SHAP values associated with age per COVID-19 measure and risk scenario (see Fig. 2). As a first observation, the overall effects tend to be smaller for scenario 1 (less severe) compared to the other risk scenarios. Aside from the findings in line with previous studies, i.e., older age is associated with higher policy support, visual inspection of the summary plots confirms heterogeneous distributions of the effects, potential nonlinear effects and effects with an opposite direction for specific measures.

Heterogeneous distributions of the effects are shown in summary plots either as clusters of SHAP values agglomerated in one or more locations or as a line of SHAP values sparsely distributed in a plot. Clusters are associated with groups of respondents with a similar effect on policy support. In contrast, sparsely distributed effects indicate differences in policy support for respondents that belong to an age group. For instance, the SHAP values associated with an advice to work from home in scenario 1 and to receive a maximum of two visitors per day in scenario 2 present three clusters of effects, with a first cluster associated with a lower effect on policy support and low age, a second cluster associated with close-to-null effects and middle age, and a third cluster associated with higher effect and older age. Sparse distributions are observed, for instance, for the advice of having maximum 2 visitors per day at home in scenario 3, or a 2G COVID-19 certificate for those who work with vulnerable people in scenario 4, where the sparse effects are associated with the extreme age groups, indicating clear differences on the policy support for such measures across respondents of the extreme age groups.

**Fig. 3:**
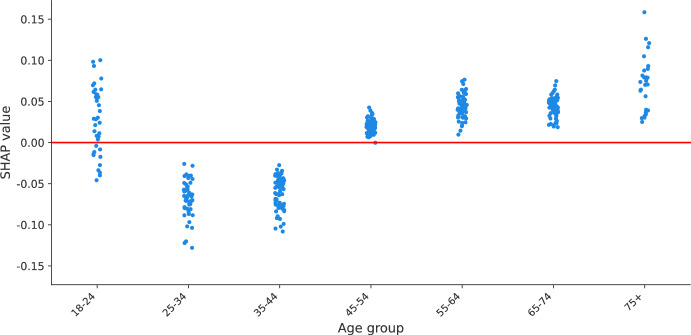
SHAP values of age of implementing a 3G COVID-19 certificate for scenario 1

Nonlinear effects are shown in summary plots as SHAP values with similar effects (i.e., close together) but associated with different age groups. An example of nonlinear effects is with the imposition of a 3G COVID-19 certificate for public transport, shows and restaurants in scenario 1. While visual inspection confirms that older age is associated with higher policy support for the measure, there is a group of points associated with middle age (coloured in purple) located in the lower tail of the plot, indicating that such respondents have low policy support comparable with respondents of the lowest age group. A scatter plot (see Fig. 3) confirms that the effect of age for implementing this measure resembles a piecewise-linear function. Age groups between 25 and 44 years old are associated with negative SHAP values, while from 45 years and older, the SHAP values are positive. The effect does not seem to be increasing or decreasing within each of the two groups but remains constant, with a jump at 45–54 years old and then remaining constant.

**Fig. 4:**
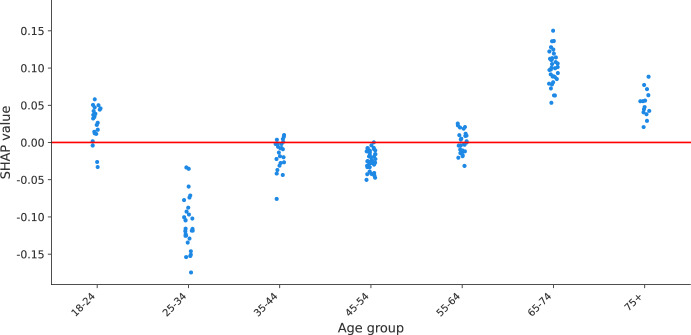
SHAP values of age of implementing a 2G COVID-19 certificate for those who work with vulnerable people in scenario 4

As another example, the SHAP values associated with imposing a COVID-19 certificate (2G) for those who work with vulnerable people in scenario 4 present a region of points around zero (no effect) and positive values associated with the lowest age. Further inspection with a scatter plot (Fig. 4) shows clear differences in the policy support for such measure per specific age group. The group of 18–24 years old has dispersed effects around zero and above. The age groups between 25 and 64 years old are associated with negative to no support, being respondents of 25–34 years old the group with the lowest support. The group of 65 years old or more are the respondents with positive support for this measure.

Finally, the effect of age on the policy support of certain measures goes in the opposite direction than expected for specific measures (recall Fig. 2). For instance, we observe that some people (points) of the lowest age groups are associated with higher policy support for advising online higher education in scenarios 3 and 4, and for closing schools in scenario 4, since these measures are likely not to affect them directly as they have lower chances of having children, compared to middle and older age groups.

#### Weight citizens' opinion compared to scientists' opinion

**Fig. 5:**
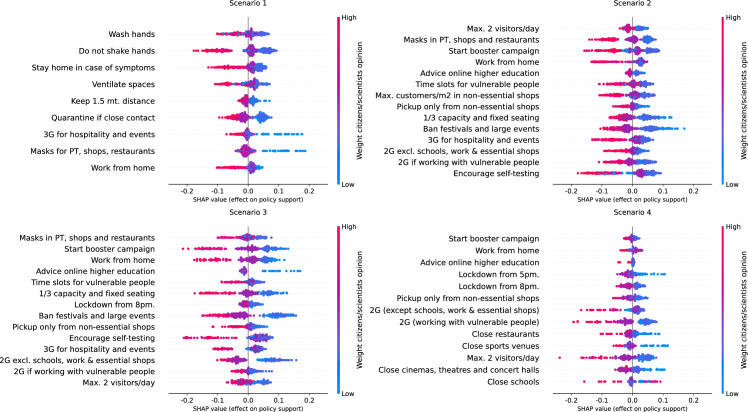
SHAP summary plots of the Weight citizens' opinion compared to scientists' opinion, per measure and risk scenario

We generate the SHAP summary plots for the Weight citizens' opinion compared to scientists' opinion per COVID-19 measure and risk scenario (see Fig. 5). As a first result, we observe that respondents who believe the government should weigh the citizens’ opinion more than the opinion of scientists are associated with lower policy support for COVID-19 measures, and vice versa for respondents who give more weight to scientists’ opinion. This result was not explored further in the previous analysis of this PVE experiment, despite this covariate being important for explaining the differences in policy support. Furthermore, SHAP summary plots evidence heterogeneous effects, either in clusters (agglomerations) of effects and sparse distributions or a combination of both.

A combination of clusters of effects and sparse distribution is observed in a summary plot as one or more groups of SHAP values associated with a specific group of covariate values (i.e., the values of the weight of citizens'/scientists' opinion), followed by a line of points associated with the rest of respondents, or vice versa. For example, consider the SHAP summary plot for the advice of working from home in scenario 2. On the one hand, respondents who believe the government should only consider citizens’ opinion are associated with lower policy support for this measure, and such effect widely differs across respondents, illustrated by the blue line of points. This result indicates strong differences in the support for this measure for respondents with the same perception about the weight the government should give to citizens’ opinion. On the other hand, for the same measure, respondents who believe the government should give more opinion to scientists’ opinion are associated with higher policy support, and they are concentrated in a single cluster, and hence they have a similar effect on the support for this measure.

#### Perceived risk of becoming sick of COVID-19

**Fig. 6:**
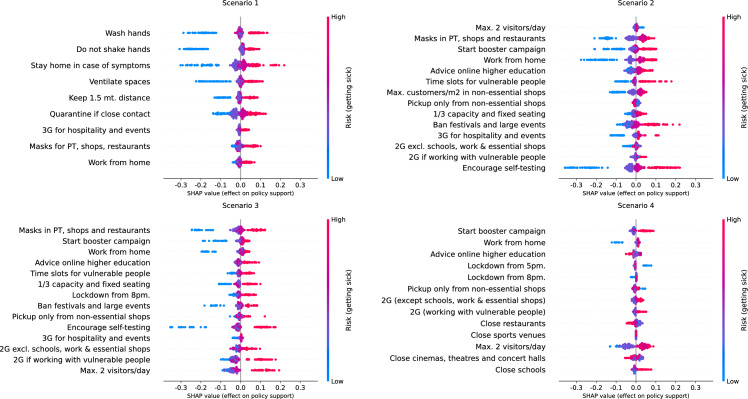
SHAP summary plots of the perceived risk of getting sick of COVID-19, per measure and risk scenario

We generate the summary plots of the perceived risk of becoming sick of COVID-19, per measure and risk scenario (see Fig. 6). As a difference with the previous covariates, sparsity of effects is more observed for respondents with a stronger opinion, i.e., with the highest and lowest perceived risk of becoming sick of COVID-19. In contrast, respondents with a moderate opinion are concentrated in a cluster close to the origin. As expected, the range of SHAP values is higher in scenarios 1, 2 and 3 since this covariate was one of the most important, whereas for scenario 4, the range of SHAP values is considerably shorter. Nevertheless, further inspection of SHAP values per scenario confirms differences in the importance of this covariate between specific measures in the same scenario. For instance, in scenario 2, for imposing mandatory masks, starting a booster campaign, working from home and encouraging self-testing, the range of SHAP values is considerably higher than for the rest of the measures in the same scenario. This is a sign that, for these measures, the perceived risk of getting sick of COVID-19 is of considerably higher importance than for the other measures in this scenario.

### Explaining policy support of specific respondent profiles with waterfall plots

To illustrate the how SHAP values explain the policy support at the respondent level, we present two waterfall plots based on two citizen profiles based on the test sample, namely Profile A and Profile B (Table 7). The selection criterion was based on four covariates: gender, age, education level and city size, in order to show clear differences between both types of respondents. In case of two or more observations of the test sample did fit with the selection criterion, the selected individual is selected randomly among them. For illustrative purposes, we only focus on the waterfall plots associated to requesting masks in public transport, shops, and restaurants under Scenario 1.

**Table 7:** Citizen profiles for waterfall plots

Covariate	Profile A	Profile B
Gender	Male	Female
Age	75 years or more	25–34 years
Education level	Low	Middle
Province	North-Brabant	South-Holland
City size	Village	Big city
Work status	Retired	Full-time
Vaccinated / Boosted	Yes / Yes	Yes / No
Risk (infected)	Moderate	High risk
Risk (getting sick)	Moderate	High risk
Risk (hospitalised)	Low risk	No risk
Risk (death)	Low risk	No risk
Weight citizens/scientists opinion	More to citizens	Only citizens
Respondent ID (from test sample)	207	502

**Fig. 7:**
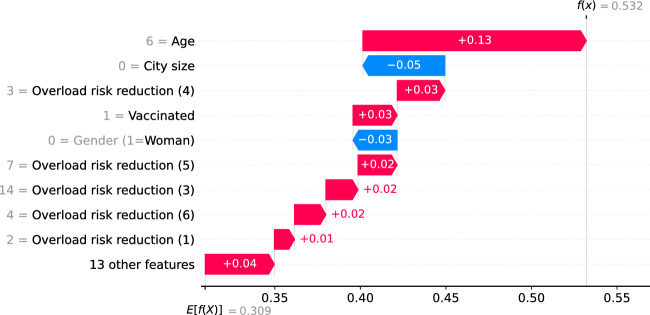
Waterfall plot of profile A for imposing a mask mandate under Scenario 1

The waterfall plot of the citizen of Profile A (respondent ID = 207) shows how his probability of choosing a mask mandate under Scenario 1 is explained by his age, city size, vaccination status, gender, and the risk reduction of other five measures (Fig. 7). For Profile A, age is the most relevant covariate and it is associated with an increase of the probability of choosing a mask mandate of 13%, the fact he lives in a village is associated with a reduction of this choice probability in a 5%, and the overload risk reduction of requesting to ventilate spaces (measure 4) of the same Scenario is associated with an 3% increase of choice probability. Other covariates that play a lower role are, for instance, vaccination status (3% increase) and his gender (3% reduction). Overall, all covariates explain a higher choice probability than the average (from 0.309 to 0.532).

**Fig. 8:**
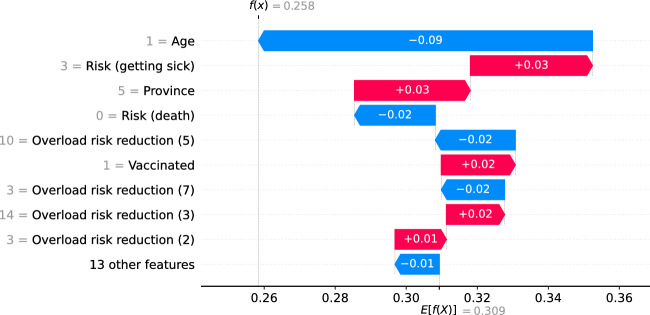
Waterfall plot for profile B for imposing a mask mandate under Scenario 1

For profile B (respondent ID = 502), her probability of choosing a mask mandate under Scenario 1 is explained by, mostly, her age, her perceived risk of getting very sick or dying of COVID-19, her living province, her vaccination status and the risk reduction of other four measures (Fig. 8). The fact this citizen is of 25–34 years old is associated with a reduction of the choice probability of 9%, her perceived risk of getting very sick of COVID-19 is associated with an increase of choice probability of 3%, and the fact she lives in the province of South-Holland is associated with a 3% probability increase. Overall, all covariates explain a lower choice probability than the average (from 0.309 to 0.258).

### Contrasting SHAP with choice modelling analysis and LCCA

The findings obtained with SHAP are contrasted with the results obtained from a choice model and LCCA for scenario 1 (see Table 8). The choice model and LCCA correspond to the models used by Mouter et al. (2022). We estimated a new version of the choice model, in which the same covariates of this study are included per COVID-19 measure, as a difference from the original study, in which only a set of constants and a single parameter for the overcrowding risk reduction were estimated. The results and the choice model are detailed in supplementary material 2. The results of the LCCA presented in this section are from Mouter et al. (2022). In this paper, only the results of scenario 1 are compared and contrasted since it is the only scenario in which the choice model converged in a reasonable amount of time (i.e., less than six hours).

**Table 8:** Contrast of interpretations between models and SHAP, scenario 1

Covariate	Choice model	LCCA (Mouter et al. 2022)	SHAP (this work)
Age group	Interpretation: older age group is associated with higher policy support (see supplementary material 2)	Interpretation: People of 65 years or more are overrepresented in the cluster that recommends all measures. Not conclusive for other age groups. Thus, heterogeneity across pre-defined groups is identified	Interpretation: Same as in choice model, with the addition of heterogeneity of effects across respondents (see Fig. 2) in the form of clusters and sparse distributions. Nonlinear effects observed (see Fig. 3)
	Significance/importance: All estimates are statistically significant, except for those associated with an advice to wash hands, not shaking hands and to stay home in case of symptoms	Significance/importance: Age is statistically significant. No information for specific measures	Significance/importance: Age is the most important covariate, on average. Age is not among the most important covariates for advising to wash hands, not shaking hands and to stay home in case of symptoms
Weight citizens'/scientists' opinion	Interpretation: More weight to citizens’ opinion is associated with lower policy support (see supplementary material 2)	Not included in the analysis	Interpretation: Same as in the choice model, with the addition of heterogeneity of effects as clusters, sparse distributions, or a combination (see Fig. 5)
	Significance/importance: All estimates are statistically significant		Significance/importance: The second-most important covariate, on average. It appears among the most important covariates across measures (see supplementary material 1)
Risk (getting sick)	Interpretation: A higher perceived risk of getting sick of COVID-19 is associated with a higher policy support (see supplementary material 2)	Not included in the analysis	Interpretation: Same as in the choice model, with the addition of heterogeneity of effects in the form of sparse distributions (see Fig. 6)
	Significance/importance: All estimates are statistically significant, except for imposing a COVID-19 certificate (3G) in the catering industry and advising working from home		Significance/importance: The third-most important, on average. This covariate is not among the most important for imposing a COVID-19 certificate (3G) in the catering industry and advising working from home
Overcrowding risk reduction	Not statistically significant in all measures, except for an advice of washing their hands	Not included in the analysis	Consistently among the least important covariates per measure, except in an advice of washing their hands

We find that SHAP reaches the same interpretations of the choice model while adding new insights in terms of heterogeneity of effects across respondents. Compared with LCCA, SHAP identifies a more detailed level of heterogeneity as the effects are computed per respondent instead of effects per pre-defined groups. For instance, we find in all models that people of the oldest age are associated with higher policy support. In SHAP, we also identify clusters of respondents with similar effects, sparse distributions of effects for respondents of a similar age and nonlinear effects that the other models do not identify. The results for the other covariates follow the same pattern: SHAP provides equivalent results to choice models and LCCA, with the addition of heterogeneity at the respondent level.

Regarding statistical significance and importance of covariates, we find that the covariates identified as the most important in SHAP coincide with the covariates identified as statistically significant in the choice model per specific COVID-19 measures. On the one hand, age group, the weight of citizens'/scientists' opinion and the perceived risk of getting very sick of COVID-19 are identified as the most important covariates on average by SHAP (see Table 5), and for each specific measure, these covariates rank on the higher part of the most important covariates per specific COVID-19 measures and at the same time they are statistically significant in the choice model (see supplementary material 1 and 2). On the other hand, the overcrowding risk reduction is ranked as the least-important covariate on average, and it ranks in the lowest positions per COVID-19 measure, coinciding with the fact that this covariate is not statistically significant in the choice model. Neither the weight of citizens'/scientists' opinion, the perceived risk of getting sick of COVID-19 nor the overcrowding risk reduction is considered in the LCCA analysis of Mouter et al. (2022).

Based on the analyses made in this paper, we compare and contrast SHAP with choice models and LCCA in four dimensions (see Table 9).

**Table 9:** Contrast of models and SHAP

Dimension	Choice model	LCCA	SHAP
Interpretation of covariates	Sign and magnitude of the estimated parameters indicate positive (negative) effect of the covariate on policy support	Probabilities per latent class characterise each predefined group in terms of sociodemographic characteristics	Sign and magnitude of SHAP values indicate the positive (negative) effect of the covariate value on the policy support per respondent
Importance of covariates	Statistical significance of parameters	Statistical significance of parameters	Magnitude of SHAP importances, compared with the other covariates
Heterogeneity of effects	Yes (observed and unobserved). Limited by the model specification	Yes (observed). Limited by the number of latent classes	Yes (observed). SHAP values are at the respondent level
Estimation time	From one hour to more than six hours	Two to three minutes	Two to three minutes

In terms of interpretation of results, we find that SHAP allows identifying the effect of covariates in the policy support in a similar way as in a choice model, with the addition of providing information at the respondent level. A similar analysis can be done with LCCA, in which the interpretation of results is made per predefined groups in terms of the probability of belonging to each of such groups. Regarding identifying the importance of covariates, both choice models and LCCA rely on identifying the statistical significance of a set of estimated parameters. In contrast, SHAP identifies the importance order of each covariate in terms of the SHAP importances.

In terms of heterogeneity, all models can capture observed (differences on effects of covariates) heterogeneity, whereas a choice model can also capture unobserved (stochastic) heterogeneity. On the one hand, SHAP is able to identify observed heterogeneity at the respondent level, thus identifying how the effects of each covariate are distributed across covariates and measures. On the other hand, choice models and LCCA can capture observed heterogeneity, but such ability is limited by the a priori model specification provided in the former, and the a priori definition of the number of latent classes in the latter. However, evaluating all possible model specifications in a choice model is time-unfeasible, whereas specifying a too high number of latent classes in LCCA can lead to a non-informative model (i.e., non-parsimonious, with few or no statistically significant parameters).

A final and practical difference between all models is the estimation time, which is critical in crises when results are needed in shorter time spans for decision-making. On the one hand, choice models are the least convenient approach, with an estimation time of around one hour for scenario 1. Furthermore, after six hours, we could not obtain convergence of the choice model for scenarios 2, 3 and 4. On the other hand, LCCA and SHAP estimation times are around three minutes for all scenarios. Considering that we show SHAP provides similar results as a choice model in the same scenario, with the addition of identifying heterogeneity of effects per covariate and measure, SHAP can be used instead of the choice model for this application.

## Discussion

In this paper, we study the factors (covariates), i.e., sociodemographic characteristics, perception indicators and experimental variables, that lead to differences in the policy support for COVID-19 measures under different risk scenarios, with a focus on how such differences are distributed across citizens. We use data from a PVE experiment to determine the citizens’ preferences for COVID-19 measures in the Netherlands (Mouter et al. 2022). We model the data with XGBoost, a ML model, and compute the SHAP values to identify the effect of each used covariate on the policy support for COVID-19 measures for each respondent of the PVE experiment. Our results show that the heterogeneity of effects on the policy support for measures can lead to considerable differences between respondents of similar profiles (e.g., age, perception) or nonlinear effects that, if neglected by only considering average effects, could lead to misinterpretation of results. Furthermore, compare and contrast SHAP with other data analysis methods, namely choice models and LCCA. We show that SHAP analysis provides similar results as conventional approaches (i.e., choice models), but with the addition of providing effects at the respondent level and in a considerably minor estimation time.

A methodological contribution is that we explored how policy makers could use the results of a SHAP analysis in their daily practices. We found that policy makers regard SHAP as a useful instrument to predict policy support among detailed subsegments of the population and also better understand (lack of) policy support. The fact that the results can be derived in two to three minutes is particularly useful in the context of COVID-19 decision-making where all decisions need to be taken under high time pressure.

### Main findings

First, we show how the policy support for COVID-19 measures is distributed across respondents in terms of the age group of respondents, the weight they believe the government should give to the opinion of citizens compared to the opinion of scientists, and the perceived risk of becoming sick of COVID-19, which are the covariates identified as with the highest importance by SHAP importances (see Table 6). Aside from confirming the findings of previous studies, including the first analysis of the PVE experiment (Mouter et al. 2022), we identify clusters of different types of respondents but with similar policy support, sparse distributions of effects for respondents with similar characteristics, effects in the opposite direction for specific measures and nonlinear effects for specific groups of respondents. For instance, we find that for closing schools in a high-risk scenario (scenario 4), respondents of the lowest age group are associated with higher policy support for the measure than respondents of other age groups, going in an opposite direction to the “average” interpretation for the rest of measures (see Fig. 2). As another example, we find that the policy support for implementing a COVID-19 certificate in scenario 1 across different age groups is a piecewise-linear function, with a negative effect for groups less than 45 years old and a positive effect for older groups (see Fig. 3). Similar findings are made for the weight of citizens'/scientists' opinion and perceived risk of getting sick of COVID-19, where combinations of clusters and sparse distributions of effects are found for specific measures and scenarios (see Figs. 5 and 6). Additionally, we show specific illustrations on how SHAP values can be used to explain the policy support of specific individuals, by using two citizen profiles and waterfall plots (Figs. 7 and 8).

Second, we show that SHAP analysis delivers the same interpretation results and identification of important covariates as a conventional choice model, with the addition of providing how the effects are distributed at the respondent level (see Tables 8 and 9), whereas contrasted with an LCCA, SHAP provides a deeper level of heterogeneity as there is no need of pre-defining a number of latent classes. The visualisation of SHAP values allows determining that older age, a higher weight to the opinion of scientists and a higher perceived risk of getting sick of COVID-19 are associated with higher policy support for COVID-19 measures, with a similar conclusion obtained from interpreting the estimated parameters of the choice model (see Table 8). Furthermore, SHAP values also provide information about how the effects are distributed across respondents, allowing for a more nuanced analysis per covariate, measure and risk scenario. Finally, we argue in favour of using SHAP for interpreting results and identifying importance, as this method provides the same results as a choice model in a considerably shorter time: two to three minutes contrasted with one to more than six hours (see Table 9).

### Policy implications

SHAP analysis can help policymakers understand which types of citizens are the most (least) reluctant to specific measures in greater detail than previous methods (i.e., choice models and LCCA) and tailor measures to increase policy support. For instance, as we found that negative support for a COVID-19 certificate in a low risk scenario (scenario 1) is concentrated in citizens 45 years old or less (see Fig. 3), policymakers can build information campaigns focused on such age groups to increase support for this measure. As another example, since we found that respondents of the middle and high age groups are associated with lower policy support for closing schools in a high-risk scenario (scenario 4, Fig. 2), policymakers can focus on such age groups to prepare compensation packages, since at the same time these groups are more likely to have children in school age than citizens of the lowest age group (i.e., below 25 years old). And, as we see that citizens who think that they have a low chance of getting sick from COVID-19 particularly dislike measures such as the advice to not shake hands and self-testing it is important to tailor communication to this group and explain the importance of the measure to people who think that they have a low chance of getting sick from COVID-19.

### Considerations and research directions

We identify a few considerations in this paper. First, our findings are bounded by the population context, the moment the sample was collected and the use of PVE as an elicitation framework. Therefore, the findings of this paper should not be extrapolated for other countries or other moments of the pandemic, even though our findings align with previous studies regarding policy support for COVID-19 measures (Sicsic et al. 2022). Second, it is relevant to notice that neither XGBoost or SHAP establish causal relationships per se (e.g., if age is higher, then policy support is higher and not vice versa). In consequence, our approach only allows policymakers to safely identify associations between covariates and the policy support for COVID-19 measures. We strongly recommend to contrast the findings from SHAP with more structural methods, such as choice models, as we did in the present work. Finally, SHAP has a longer computation time than alternative explanation methods (e.g., LIME, LRP), often in the order of minutes at the minimum. Hence, researchers and policymakers should carefully assess the advantages of SHAP (i.e., built in solid theory, global and local explanations) in light of its computational demands, particularly when the urgency of obtaining results is a priority.

Finally, as a further research direction, we envision using SHAP to further explain the policy support for measures for specific profiles of respondents. This paper did only cover this direction for two examples since the range of possible profiles to explore is unfeasible to cover in a manuscript. To overcome this, developing a consultation (web-based) platform to build specific queries is possible. The interested analyst can construct specific profiles of citizens from a previously trained ML model and obtain their specific set of SHAP values as a result. Policymakers could benefit from such a web-based platform by counting with information about the policy support for COVID-19 measures for different individuals, different measures, and scenarios in a fine-grained level of detail.

## Supplementary Information

Below is the link to the electronic supplementary material.

Supplementary file1 (DOCX 18970 KB)

## Data Availability

Data will be available upon publication in the 4TU.ResearchData repository.

## Footnotes

1 Furthermore, no considerable model fit improvements were found with alternative ML models in preliminary tests.
