# Supplementary material for: Using XGBoost and SHAP to explain citizens’ differences in policy support for reimposing COVID-19 measures in the Netherlands
Source: Qual Quant. 2024 Jul 24;59(1):381–409. doi: 10.1007/s11135-024-01938-2 (PMC11929738; doi:10.1007/s11135-024-01938-2)
Supplement: Supplementary file 1 — Supplementary file1 (DOCX 18970 KB) [file 11135_2024_1938_MOESM1_ESM.docx]

**Using XGBoost and SHAP to explain citizens’ differences in policy support for reimposing COVID-19 measures in the Netherlands**

**Supplementary material**

**Supplementary material 1: SHAP summary plots per risk scenario**

**Fig. 1** SHAP summary plots per COVID-19 measure, scenario 1

**Fig. 2** SHAP summary plots per COVID-19 measure, scenario 2

**Fig. 3** SHAP summary plots per COVID-19 measure, scenario 3

**Fig. 4** SHAP summary plots per COVID-19 measure, scenario 4

**Supplementary material 2: Description and estimation results of the choice model**

The choice model employed to contrast the findings of SHAP is an extended version of the portfolio choice model employed by Mouter et al. (2022). The version employed in our work includes the same set of covariates employed in the SHAP analysis as explanatory variables per COVID-19 measure, in order to allow a contrast between both analysis methods. The model used by Mouter et al. (2022) only considered the sum of overcrowding risk reductions of the selected package of measures by the respondents.

The portfolio choice model was initially proposed by Bahamonde-Birke & Mouter (2019). This model is based on the Random Utility Model (RUM) employed to analyse discrete choice data, extended to consider that respondents can choose packages of alternatives instead of a single (discrete) alternative.

In the portfolio choice model, respondents seek to maximise their utility derived from their chosen combination of alternatives, and hence, higher utility is associated with higher policy support. In turn, the utility of each alternative is a function of their experimental features (i.e., attributes) and individual-specific covariates (e.g., sociodemographic characteristics, perception indicators). Specifically, the utility of respondent $n$ for a combination of alternatives $p$ is given by equation (A1):

| $U_{np}=V_{np}+\varepsilon_{np}=\sum_{j=1}^{J} y_{nj}\cdot(\delta_{j}+\beta'X_{nj}+\theta_{j}'Z_{n})+\varepsilon_{np},$ | (A1) |
| --- | --- |

Where $y_{nj}$ is a binary variable equal to one if the respondent choose alternative $j$, $X_{nj}$ is a vector of characteristics of the alternative $j$, (e.g., overcrowding risk reductions in the PVE experiment), $Z_{n}$ is a vector of individual-specific covariates of respondent $n$, $\delta_{j}$, $\beta$ and $\theta_{j}$ are parameters to be estimated, and $\varepsilon_{np}$ is a stochastic error with a Gumbel distribution. Under these assumptions, the probability of choosing a combination of alternatives $p$ take the form of a multinomial logit (MNL) model, as described by equation (A2):

| $p(U_{np}\geq U_{nq}, \forall q\neq p)=exp(V_{np})/\sum_{q} exp(V_{nq})$ | (A2) |
| --- | --- |

The estimated parameters $\delta_{j}$, $\beta$ and $\theta_{j}$ have an economic interpretation. Firstly, $\delta_{j}$ are alternative-specific constants interpreted as the utility increase when their associated alternative is chosen. Secondly, the sign of $\beta$ is interpreted as the contribution of an attribute increase to the respondents utility. If $\beta$ is positive, then increases on its associated attribute generate increases on the respondent’s utility, and if $\beta$ is negative, then the increase of its associated attribute generates a decrease of the respondent’s utility. Lastly, the sign of $\theta_{j}$ is interpreted as the effect of the individual-specific covariates on the respondent’s utility. If $\theta_{j}$ is positive, the associated covariate $Z$ induces increases in the utility, while if If $\theta_{j}$ is negative, the covariate is associated with decreases in the utility.

*Table A1: Estimation results of the choice model, scenario 1 (continues in the next page)*

|  | Advice to wash hands | Advice to not shake hands | Advice to stay home in case of symptoms | Advice to ventilate spaces | Advice to keep 1.5 mt. distance | Advice of quarantine if close contact | COVID-19 certificate (3G) for hospitality industry | Wear masks in PT, shops & restaurants | Advice to work from home |
| --- | --- | --- | --- | --- | --- | --- | --- | --- | --- |
| Measure-specific constant | **-0.780^**^** | **-1.062^***^** | **-0.676^*^** | -0.372 | **-0.937^**^** | **-1.078^***^** | **-2.161^***^** | **-1.701^***^** | **-0.852^**^** |
|  | **(0.246)** | **(0.320)** | **(0.304)** | (0.257) | **(0.288)** | **(0.278)** | **(0.373)** | **(0.273)** | **(0.276)** |
| Overload risk reduction | **0.140^*^** | 0.064 | -0.004 | -0.019 | 0.015 | -0.020 | 0.029 | 0.011 | 0.024 |
|  | **(0.061)** | (0.059) | (0.020) | (0.029) | (0.019) | (0.029) | (0.066) | (0.031) | (0.058) |
| Is a woman | 0.196 | **0.262^*^** | 0.171 | 0.198 | 0.102 | **0.324^**^** | 0.217 | 0.158 | 0.158 |
|  | (0.108) | **(0.105)** | (0.106) | (0.104) | (0.103) | **(0.104)** | (0.118) | (0.110) | (0.103) |
| Middle age | 0.075 | 0.178 | -0.040 | 0.165 | 0.267^*^ | **0.291^*^** | 0.202 | -0.036 | 0.224 |
|  | (0.126) | (0.122) | (0.123) | (0.121) | (0.118) | **(0.119)** | (0.133) | (0.125) | (0.118) |
| Higher age | -0.041 | -0.171 | 0.171 | 0.040 | 0.122 | 0.295 | 0.366 | **0.646^**^** | 0.196 |
|  | (0.239) | (0.229) | (0.236) | (0.230) | (0.224) | (0.226) | (0.240) | **(0.231)** | (0.222) |
| Middle education | 0.191 | 0.073 | 0.190 | 0.087 | -0.107 | -0.034 | -0.124 | **-0.288^*^** | 0.004 |
|  | (0.131) | (0.128) | (0.129) | (0.127) | (0.125) | (0.127) | (0.141) | **(0.132)** | (0.126) |
| Higher education | **0.333^*^** | 0.216 | 0.163 | **0.353^**^** | -0.185 | -0.020 | -0.267 | **-0.407^**^** | 0.125 |
|  | **(0.139)** | (0.134) | (0.135) | **(0.134)** | (0.131) | (0.132) | (0.149) | **(0.138)** | (0.131) |
| Friesland | 0.197 | -0.002 | 0.127 | 0.139 | 0.139 | 0.204 | -0.223 | -0.003 | -0.192 |
|  | (0.184) | (0.177) | (0.182) | (0.180) | (0.175) | (0.176) | (0.203) | (0.191) | (0.176) |
| Gelderland | 0.165 | 0.088 | -0.324 | -0.119 | 0.096 | -0.037 | -0.155 | 0.211 | 0.259 |
|  | (0.228) | (0.221) | (0.221) | (0.220) | (0.219) | (0.223) | (0.253) | (0.233) | (0.218) |
| Groningen | -0.158 | 0.083 | -0.354 | -0.447 | 0.412 | 0.239 | 0.166 | -0.077 | -0.570 |
|  | (0.331) | (0.331) | (0.327) | (0.325) | (0.327) | (0.328) | (0.353) | (0.359) | (0.340) |
| Limburg | 0.234 | 0.255 | 0.256 | 0.116 | 0.402 | **0.474^*^** | 0.007 | 0.130 | 0.137 |
|  | (0.221) | (0.214) | (0.219) | (0.213) | (0.209) | **(0.211)** | (0.234) | (0.223) | (0.208) |
| North Brabant | 0.293 | 0.151 | 0.034 | 0.056 | 0.294 | 0.189 | -0.126 | 0.299 | 0.117 |
|  | (0.162) | (0.156) | (0.158) | (0.157) | (0.154) | (0.156) | (0.176) | (0.165) | (0.153) |
| North Holland | 0.360 | 0.368 | 0.050 | 0.066 | 0.346 | 0.275 | 0.044 | **0.614^*^** | 0.080 |
|  | (0.262) | (0.253) | (0.251) | (0.248) | (0.243) | (0.245) | (0.275) | **(0.252)** | (0.243) |
| Utrecht | 0.482 | 0.118 | 0.297 | -0.063 | 0.466 | 0.486 | 0.396 | 0.164 | 0.365 |
|  | (0.299) | (0.276) | (0.286) | (0.275) | (0.274) | (0.275) | (0.293) | (0.293) | (0.273) |
| Overijssel | **0.386^*^** | 0.321 | 0.178 | -0.046 | 0.247 | **0.357^*^** | -0.249 | 0.031 | -0.110 |
|  | **(0.181)** | (0.174) | (0.175) | (0.172) | (0.169) | **(0.170)** | (0.195) | (0.182) | (0.169) |
| Zeeland | -0.056 | **-0.415^*^** | 0.214 | -0.034 | 0.221 | 0.209 | 0.112 | 0.102 | -0.160 |
|  | (0.211) | **(0.208)** | (0.214) | (0.208) | (0.206) | (0.208) | (0.230) | (0.223) | (0.207) |
| South Holland | -0.136 | 0.107 | -0.310 | 0.336 | -0.034 | 0.149 | -0.282 | -0.491 | 0.352 |
|  | (0.277) | (0.278) | (0.276) | (0.284) | (0.276) | (0.277) | (0.332) | (0.325) | (0.274) |
| Observations | 1888 |  |  |  |  |  |  |  |  |
| Log-likelihood | -10,803.28 |  |  |  |  |  |  |  |  |
| **Notes:** Standard errors are in parenthesis. Statistically significant estimates (up to 95% confidence level) are in **bold**. ***p < 0.001; **p < 0.01; *p < 0.05 | | | | | | | | | |

**Table A1 (continuation)**

|  | Advice to wash hands | Advice to not shake hands | Advice to stay home in case of symptoms | Advice to ventilate spaces | Advice to keep 1.5 mt. distance | Advice of quarantine if close contact | COVID-19 certificate (3G) for hospitality industry | Wear masks in PT, shops & restaurants | Advice to work from home |
| --- | --- | --- | --- | --- | --- | --- | --- | --- | --- |
| Medium city | 0.003 | -0.125 | 0.079 | **-0.259^*^** | -0.239 | -0.090 | 0.089 | -0.146 | -0.159 |
|  | (0.138) | (0.133) | (0.136) | **(0.132)** | (0.131) | (0.132) | (0.150) | (0.142) | (0.131) |
| Big city | -0.073 | -0.090 | -0.160 | **-0.244^*^** | -0.070 | -0.086 | 0.117 | -0.013 | -0.146 |
|  | (0.114) | (0.110) | (0.111) | **(0.109)** | (0.107) | (0.109) | (0.123) | (0.115) | (0.108) |
| Incapacitated | 0.217 | 0.110 | 0.231 | 0.403^**^ | 0.099 | 0.079 | -0.037 | -0.108 | -0.114 |
|  | (0.147) | (0.142) | (0.144) | (0.142) | (0.139) | (0.140) | (0.163) | (0.153) | (0.139) |
| Retired | 0.295 | 0.090 | 0.249 | 0.381 | -0.005 | -0.057 | -0.124 | -0.207 | -0.241 |
|  | (0.232) | (0.222) | (0.226) | (0.222) | (0.217) | (0.219) | (0.253) | (0.239) | (0.220) |
| Housewife -husband | 0.396 | 0.303 | 0.136 | 0.456 | 0.141 | 0.064 | -0.224 | -0.209 | 0.157 |
|  | (0.254) | (0.243) | (0.250) | (0.244) | (0.237) | (0.239) | (0.257) | (0.245) | (0.236) |
| Not working | 0.209 | 0.171 | -0.133 | 0.359 | 0.181 | 0.023 | -0.025 | 0.171 | 0.082 |
|  | (0.191) | (0.185) | (0.184) | (0.183) | (0.180) | (0.182) | (0.206) | (0.190) | (0.180) |
| Student | 0.434 | -0.146 | **0.602^*^** | -0.052 | 0.245 | 0.380 | 0.219 | 0.163 | -0.049 |
|  | (0.279) | (0.253) | **(0.272)** | (0.250) | (0.250) | (0.251) | (0.290) | (0.271) | (0.252) |
| Vaccinated | **0.446^**^** | 0.407^*^ | **0.483^**^** | 0.317 | **0.482^**^** | 0.210 | **0.658^**^** | **0.762^***^** | **0.347^*^** |
|  | **(0.169)** | (0.167) | **(0.166)** | (0.166) | **(0.169)** | (0.171) | **(0.226)** | **(0.203)** | **(0.170)** |
| Boosted | 0.110 | 0.222 | 0.318^*^ | 0.036 | -0.003 | 0.195 | 0.248 | 0.118 | 0.122 |
|  | (0.137) | (0.131) | (0.132) | (0.132) | (0.130) | (0.131) | (0.153) | (0.140) | (0.130) |
| High risk (infected) | 0.032 | 0.098 | -0.111 | 0.042 | **-0.291^*^** | -0.102 | -0.026 | -0.058 | -0.061 |
|  | (0.122) | (0.116) | (0.119) | (0.117) | **(0.115)** | (0.116) | (0.131) | (0.122) | (0.114) |
| High risk (getting sick) | 0.078 | **0.282^*^** | **0.407^**^** | 0.148 | **0.285^*^** | **0.325^*^** | 0.047 | 0.027 | 0.197 |
|  | (0.138) | **(0.132)** | **(0.135)** | (0.132) | **(0.129)** | **(0.130)** | (0.148) | (0.139) | (0.129) |
| High risk (hospitalised) | -0.029 | -0.106 | -0.064 | -0.110 | -0.114 | 0.052 | -0.239 | **0.356^*^** | -0.126 |
|  | (0.179) | (0.172) | (0.175) | (0.171) | (0.166) | (0.168) | (0.192) | **(0.173)** | (0.166) |
| High risk (death) | -0.253 | **-0.382^*^** | -0.182 | **-0.424^*^** | -0.021 | -0.164 | 0.270 | -0.118 | -0.175 |
|  | (0.180) | **(0.173)** | (0.176) | **(0.172)** | (0.168) | (0.170) | (0.193) | (0.175) | (0.169) |
| Higher weight to scientists opinion | **0.255^*^** | **0.367^***^** | **0.295^**^** | **0.253^*^** | **0.233^*^** | **0.382^***^** | **0.290^**^** | **0.249^*^** | 0.106 |
|  | **(0.107)** | **(0.102)** | **(0.104)** | **(0.102)** | **(0.099)** | **(0.100)** | **(0.112)** | **(0.105)** | (0.099) |
| Observations | 1888 |  |  |  |  |  |  |  |  |
| Log-likelihood | -10,803.28 |  |  |  |  |  |  |  |  |
| **Notes:** Standard errors are in parenthesis. Statistically significant estimates (up to 95% confidence level) are in **bold**. ***p < 0.001; **p < 0.01; *p < 0.05 | | | | | | | | | |

**References**

Bahamonde-Birke, F. J., & Mouter, N. (2019). *About positive and negative synergies of social projects: Treating correlation in participatory value evaluation.*

Mouter, N., Jara, K. T., Hernandez, J. I., Kroesen, M., de Vries, M., Geijsen, T., Kroese, F., Uiters, E., & de Bruin, M. (2022). Stepping into the shoes of the policy maker: Results of a Participatory Value Evaluation for the Dutch long term COVID-19 strategy. *Social Science & Medicine*, *314*, 115430. DOI: 10.1016/j.socscimed.2022.115430
